# Water-saving guard cell-mesophyll cell model captures temporally differential enzymatic and transporter activities during C_3_–crassulacean acid metabolism transition

**DOI:** 10.1093/plphys/kiaf048

**Published:** 2025-02-21

**Authors:** Devlina Sarkar, Sudip Kundu

**Affiliations:** Department of Biophysics, Molecular Biology and Bioinformatics, University of Calcutta, Kolkata-700009, India; Department of Biophysics, Molecular Biology and Bioinformatics, University of Calcutta, Kolkata-700009, India

## Abstract

Gradual reduction of transpirational water loss captures differential metabolic enzyme and transporter activities in guard and mesophyll cells during the C_3_-to-crassulacean acid metabolism transition.

Dear Editor,

CAM (crassulacean acid metabolism) plants reduce the water loss through transpiration in arid environments (Wickell et al. 2021), using an alternative pathway of carbon assimilation. To ensure food security, engineering CAM into C_3_ plants can be achieved by inverting the stomatal rhythm and the timing of major CO_2_ uptake from day to night. Identification of the metabolic enzymes and intra-cellular transporters, present in both C_3_ and CAM but having different differential temporal activities throughout the diel cycle (Yang et al. 2015; Heyduk et al. 2019) and quantitative estimations of the flux distributions along the biochemical trajectory of C_3_-to-CAM transition, may help us to achieve the goal. Here, we simulate a constraint-based combined metabolic model of guard cell (GC) and mesophyll cell (MC), linking temporal fluctuations of temperature (T) and relative humidity (RH) throughout the diurnal cycle with osmolyte accumulation dependent stomatal opening, CO_2_ uptake, and transpirational water loss. Starting with C_3_ metabolism, gradual increase in water-use efficiency (WUE) captures several known and previously unknown differential activities of metabolic enzymes, transporters, sugar-malate cycle, etc. in GC and MC during C_3_-to-CAM transition.

Previous computational efforts compared the MC metabolisms of C_3_ and CAM plants (Cheung et al. 2014), explored the potential metabolic C_3_-to-CAM transition of MC by declining environmental CO_2_ uptake at daytime (Tay et al. 2021), analyzed metabolic changes in MC during C_3_-to-CAM transition by reducing transpirational water loss while compromising phloem output (Töpfer et al. 2020), and studied the C_3_ GC metabolism (Tan and Cheung 2020). However, metabolisms of GC and MC are interdependent. The stomatal pores, regulated by the osmolytes accumulation in GC, control the transpirational water loss and the uptake of CO_2_. While this CO_2_ is the source of carbon for both GC–MC metabolisms, MC supplies sucrose, one of the osmolytes, balancing the osmotic pressure in GC. Thus, investigating the changes in this coupled metabolism as WUE increases is particularly important. We have already reported a 6-phase combined metabolic model of GC and MC (Sarkar and Kundu 2024) where the 6 phases are named as phase 1 to phase 6 (abbreviated as P1 to P6). Phases 1 to 3 represent the 3 phases of day—dawn (0 to 1 h), mid-day (1 to 11 h), and afternoon (11 to 12 h), respectively; and phases 4 to 6 represent the 3 phases of night—dusk (12 to 13 h), midnight (13 to 23 h), and end of night (23 to 24 h), respectively. In this work, the model has been modified to link the transpirational water loss by the system with the CO_2_ demand, T, and RH using a previously used gas diffusion model (Töpfer et al. 2020). Furthermore, to include the occurrence of many (270 to 400) MCs per GC in a leaf (Reckmann et al. 1990), we consider an intermediate value. Firstly, when we maximize the WUE of the model with C_3_ constraints, results indicate the switching of the C_3_ metabolism to CAM. For example, the model starts taking CO_2_ at night; and the nighttime malate storage and the activities of some cytosolic enzymes related to CAM, such as PEPC (phosphoenolpyruvate carboxylase), PEPCK (phosphoenolpyruvate carboxykinase), and MDH (malate dehydrogenase), increase. Then, we gradually decrease the total water loss throughout the diel cycle and simulate the model using a 2-step iteration method to reach to CAM from C_3_ (Fig. 1A, Supplementary Text S1, Supplementary Figs. S1 and S2, Supplementary Table S1, Supplementary Data Sets 1, A and B and S2). The increased WUE leads to gradual but nonlinear decrease in daytime CO_2_ uptake, increase in daytime starch, and nighttime malate storage in both GC and MC. The fluxes through the reactions transporting starch from phase 3 to 4 (daytime storage) and malate from phase 6 to 1 (nighttime storage) are presented in Fig. 1B (Supplementary Text S2, Supplementary Data Set 3). Results capture the temporal differences in accumulation of osmolytes like K^+^ (Fig. 1C, Supplementary Text S3, Supplementary Data Set 3) and distinct metabolic behaviors of cells even between different phases of day and night. We observe a sharp decline in the total water loss during the transition from C_3_ (13,42,630 µmol·m^−2^·s^−1^) to CAM (1,80,278 µmol·m^−2^·s^−1^). And an intermediate point of the transition, with a total water loss of 7,80,158 µmol·m^−2^·s^−1^, is considered as an intermediate species. Temporally differential flux distributions of some enzymatic reactions, characteristics of C_3_–CAM transition, are presented in Fig. 1, D to I (Supplementary Text S4, Supplementary Data Set 3). During the transition, phases 5 and 6 show increased activities of cytosolic PEPC, MDH, and glycolytic enzymes, while phases 1 and 2 exhibit prominent activities of PEPCK, ME, and enzymes related to gluconeogenesis and starch production in both the cells (Fig. 1, D to I). The observed higher starch storage in GC compared to MC at daytime is also observed in a previous study (Castano 2020). The enzymatic reactions and transporters having flux values highly correlated with increasing WUE involve different metabolic pathways such as Calvin cycle, gluconeogenesis, glycolysis, TCA cycle, pentose-phosphate pathway (PPP), nitrogen assimilation pathway, mitochondrial and plastidial electron transport chain (ETC), carboxylation–decarboxylation, and plastidial-cytosolic and mitochondrial-cytosolic shuttles; and interestingly, the similar patterns for some of them are observed in experimental studies on ice plant by introducing salinity or draught stresses (Kong et al. 2020; Guan et al. 2021). The up- and downregulated activities of enzymes of abovementioned pathways throughout the diel cycle are shown in Fig. 2 (Supplementary Text S4, Supplementary Data Set 3). The results presented here are for malate contributing 90% of the negative charge required to balance the positive charge of K^+^. We observe similar results within a range of 46% to 100% in comparison with Santelia and Lawson 2016, which reported that malate can contribute between 50% and 90% as a counterion for K^+^. Below the lower limit of malate's contribution, PEPC exhibits higher activity in MC than in GC during daytime in C_3_ plants, which is unlikely to happen (Daloso et al. 2017). The simulation using T and RH of a very hot and dry Indian region, Jaipur, indicates that the transpirational water loss varies during C_3_-to-CAM transition, but the pattern remains almost same (Supplementary Text S4). In summary, our time-resolved combined model of GC and MC allows us to infer that besides MC, GC also has its own pattern of shifting the metabolism from C_3_ to CAM and same set of enzymes has different temporal activities in the 2 cells throughout the diel cycle. Although, many physiological and anatomical differences in C_3_ and CAM, like increased succulence in leaf and stem tissues, signaling pathways regulating stomatal rhythm, etc. (Griffiths 1989; Nelson et al. 2005), are not included in this study, the identification of temporally differential flux pattern of key metabolic pathways and their quantitative estimations add an important step toward engineering CAM into C_3_ plants.

**Figure 1.**
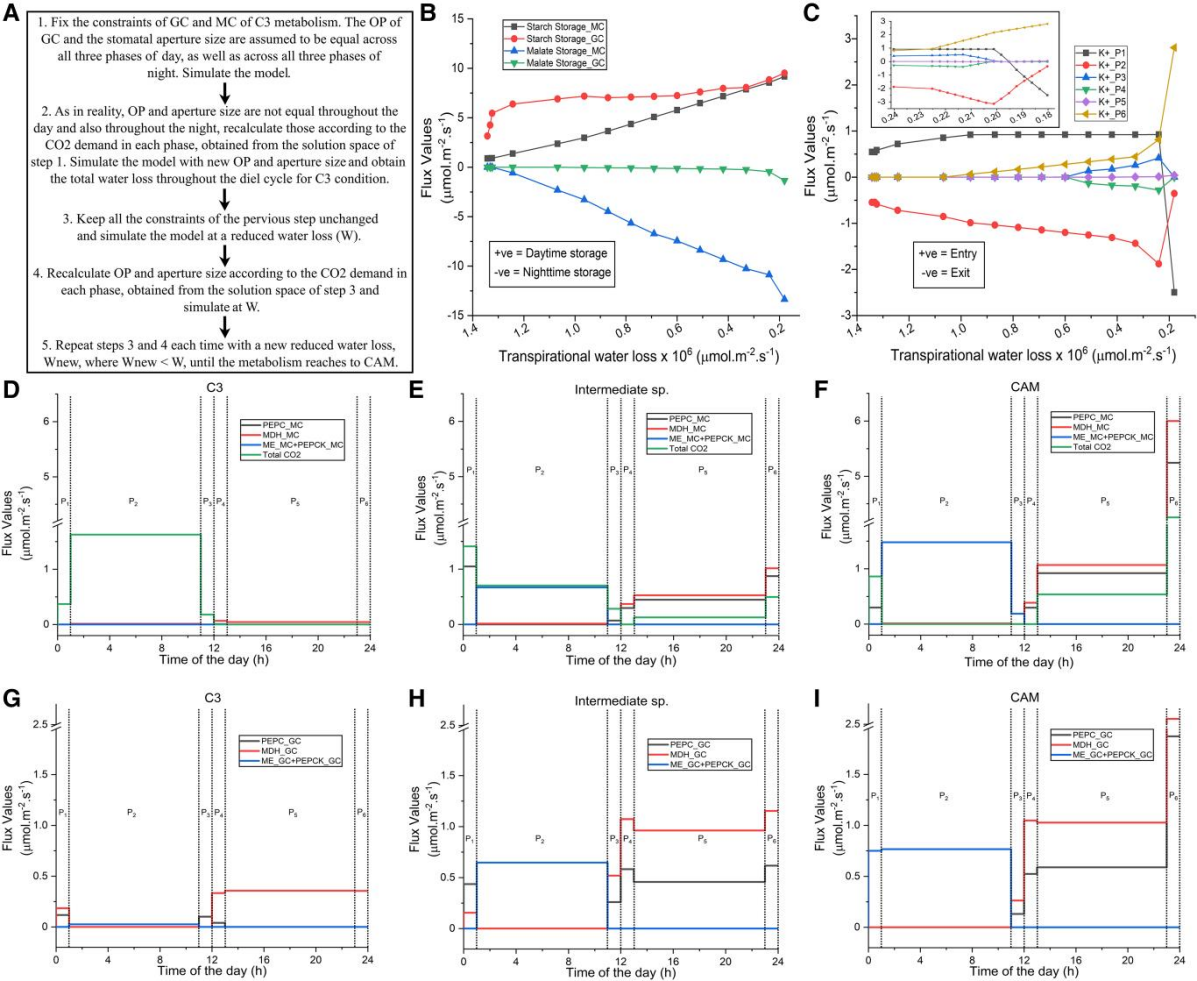
Reduced transpirational water loss forces C_3_-to-CAM transition. **A)** The flowchart of the method to simulate C_3_–CAM transition by reducing water loss. **B)** The changes in the storages of starch and malate in GC and MC throughout the C_3_–CAM transition (high to low transpirational water loss) and **C)** the variations in K^+^ uptake and release in GC throughout the transition are shown. Temporal variations of total CO_2_ uptake, activities of PEPC, cytosolic MDH, and sum of ME and PEPCK in MC of C_3_, an intermediate species, and CAM are shown in **D)**, **E)**, and **F)**, respectively. The activities of these enzymes in GC of C_3_, an intermediate species, and CAM are shown in **G)**, **H)**, and **I)**, respectively. For C_3_ and CAM, the total water loss for 1 GC and 300 MCs is 13,42,630 and 1,80,278 µmol·m^−2^·s^−1^, respectively. An intermediate point of the transition, having a total water loss of 7,80,158 µmol·m^−2^·s^−1^, represents an intermediate species. In **B)** and **C)**, transpirational water loss is presented for 300 MCs and 1 GC. Dotted lines represent the separation of the phases (for details, see Supplementary Texts S1 to S4). “x_GC” and “x_MC” represent the activities of enzyme x in GC and MC, respectively. GC, guard cell; MC, mesophyll cell; CAM, crassulacean acid metabolism; MDH, malate dehydrogenase; ME, malic enzyme; PEPC, phosphoenolpyruvate carboxylase; PEPCK, phosphoenolpyruvate carboxykinase.

**Figure 2.**
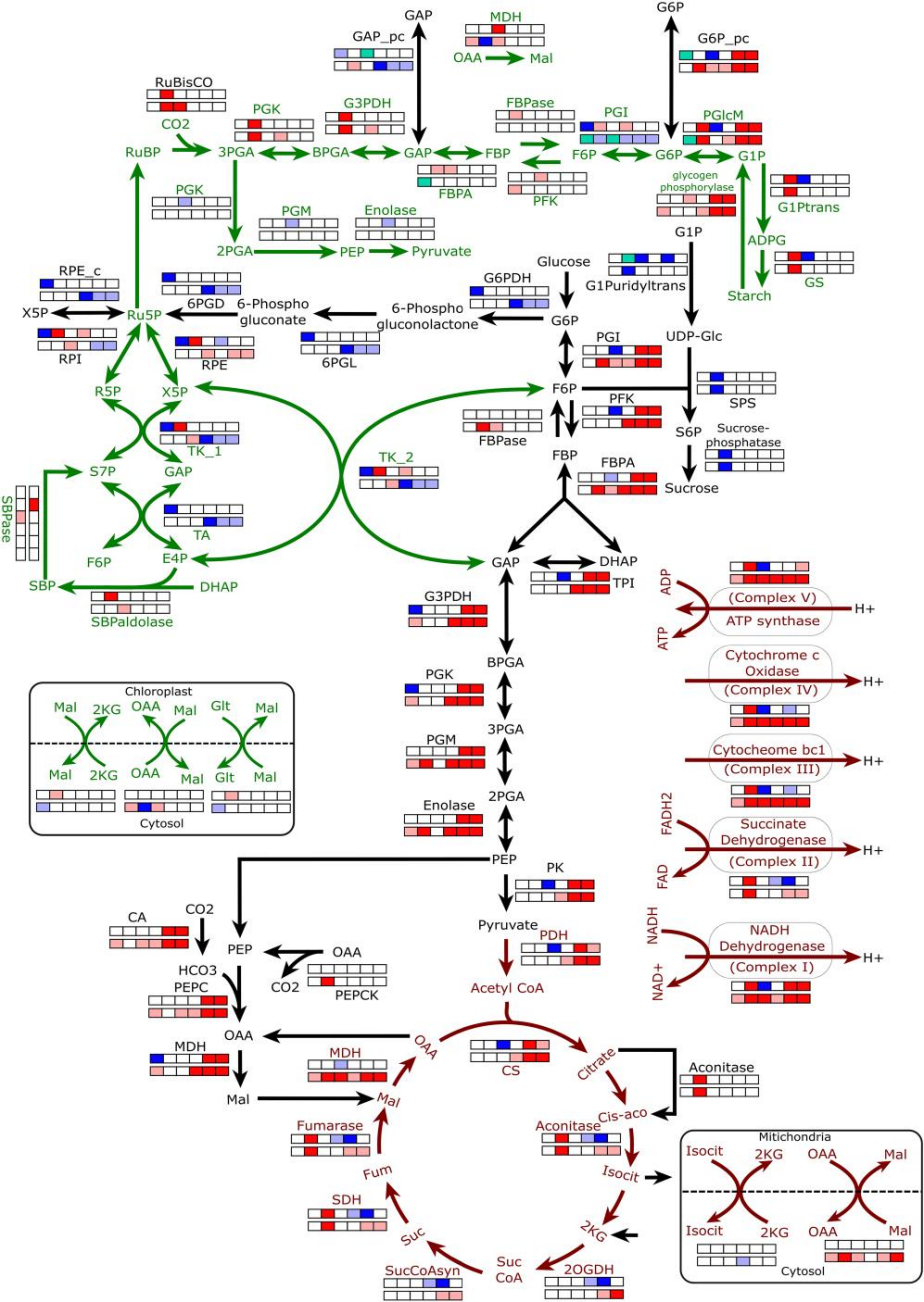
The diagram represents pathways of central carbon metabolism. For each 12-square box, the upper and lower rows (each row contains 6 boxes) represent differential activities of enzymatic reactions/transporters in 6 phases in GC and MC, respectively. Red (upregulation) and dark-blue (downregulation) represent enzymatic activities having high correlation value (>0.9) with increasing WUE, whereas pink (upregulation) and light-blue (downregulation) represent those having correlation between 0.8 and 0.9. The sea-green color represents that the reaction's direction changes during C_3_-to-CAM transition in that phase. Green, black, and brown arrows represent reactions occurring in chloroplast, cytosol, and mitochondria, respectively (for details, see Supplementary Text S4). GC, guard cell; MC, mesophyll cell; CAM, crassulacean acid metabolism; 2KG, 2-ketogluterate; 2OGDH, 2-oxogluterate dehydrogenase; 2PGA, 2-phosphoglycerate; 3PGA, 3-phosphoglycerate; 6PGD, 6-phosphogluconate dehydrogenase; 6PGL, 6-phosphogluconolactonase; BPGA, 1,3-bisphosphoglycerate; CA, carbonic anhydrase; CS, citrate synthase; DHAP, dihydroxyacetone phosphate; E4P, erythrose-4-phosphate; FBP, fructose-1,6-bisphosphate; FBPA, fructose-1,6-bisphosphate aldolase; FBPase, fructose-1,6-bisphosphatase; Fum, fumarate; G1P, glucose-1-phosphate; G6P, glucose-6-phosphate; Glc, glucose; F6P, fructose-6-phosphate; G6PDH, glucose-6-phosphate dehydrogenase; G3PDH, glyceraldehyde-3-phosphate dehydrogenase; G1Ptrans, glucose-1-phosphate adenylyltransferase; G1Puridyltrans, glucose-1-phosphate uridyltransferase; GAP, glyceraldehyde-3-phosphate; Glt, glutamate; GS, glycogen synthase; Isocit, isocitrate; Mal, malate; MDH, malate dehydrogenase; OAA, oxaloacetate; PDH, pyruvate dehydrogenase; PEP, phosphoenolpyruvate; PEPC, phosphoenolpyruvate carboxylase; PEPCK, phosphoenolpyruvate carboxykinase; PFK, phosphofructokinase; PGI, phosphoglucoisomerase; PGlcM, phosphoglucomutase; PGK, phosphoglycerate kinase; PGM, phosphoglycerate mutase; PK, pyruvate kinase; RuBP, ribulose-1,5-bisphosphate; RuBisCO, ribulose-1,5-bisphosphate carboxylase/oxygenase; RPE, ribulose-5-phosphate 3-epimerase; RPI, ribose-5-phosphate isomerase; Ru5P, ribulose 5-phosphate; R5P, ribose 5-phosphate; S7P, sedoheptulose 7-phosphate; SBP, sedoheptulose-1,7-bisphosphate; SBPase, sedoheptulose-1,7-bisphosphatase; SBPaldolase, sedoheptulose-1,7-bisphosphate aldolase; S6P, sucrose-6-phosphate; SDH, succinate dehydrogenase; SPS, sucrose-phosphate synthase; Suc, succinate; SucCoA, succinyl-CoA; SucCoAsyn, succinyl-CoA synthetase; TA, transaldolase; TK, transketolase; TPI, triose-phosphate isomerase; X5P, xylulose-5-phosphate.

## Supplementary Material

kiaf048_Supplementary_Data

## Data Availability

The model files (in excel and sbml format) and the codes are provided in Supplementary Data Sets 1, A and B and 2, respectively.

## Supplementary data

The following materials are available in the online version of this article.

**Supplementary Text S1**. Details related to the Fig. 1A.

**Supplementary Text S2**. Details related to Fig. 1B.

**Supplementary Text S3**. Details related to Fig. 1C.

**Supplementary Text S4**. Details related to Figs. 1, D to I and 2.

**Supplementary Figure S1**. Plot of the diffusion coefficient of CO_2_ ($D_{CO_{2}}$) vs temperature (T).

**Supplementary Figure S2**. Schematic representation of the model.

**Supplementary Table S1**. Constraints applied to GC and MC throughout the C_3_–CAM transition.

**Supplementary Data Set 1A**. The model file in excel format.

**Supplementary Data Set 1B**. The model file in sbml format.

**Supplementary Data Set 2**. Codes for simulating the C_3_-to-CAM continuum.

**Supplementary Data Set 3**. The excel file containing the solution space of each point in the C_3_-to-CAM continuum.

## Dive Curated Terms

The following phenotypic, genotypic, and functional terms are of significance to the work described in this paper:

MDH Gramene: AT3G47520
MDH Araport: AT3G47520

RPE Gramene: AT5G61410
RPE Araport: AT5G61410

PEPC Gramene: Sobic.010G160700
PEPC Araport: Sobic.010G160700

PEPCK Gramene: Sobic.001G432800
PEPCK Araport: Sobic.001G432800

PGK Gramene: AT3G12780
PGK Araport: AT3G12780

TPI Gramene: AT3G55440
TPI Araport: AT3G55440
